# Advances and Future Prospects of Pigment Deposition in Pigmented Rice

**DOI:** 10.3390/plants14060963

**Published:** 2025-03-19

**Authors:** Hong Lang, Xingtian Jia, Bing He, Xiaoming Yu

**Affiliations:** 1 School of Agriculture, Jilin Agricultural Science and Technology University, Jilin 132101, China; hebing02358531@126.com (B.H.); yuxm446@nenu.edu.cn (X.Y.); 2 Tongliao Institute of Agricultural and Animal Husbandry Sciences, Tongliao 028000, China; tlsnys24002300@163.com

**Keywords:** pigmented rice, metabolites, genetic basis, regulatory genes

## Abstract

Pigmented rice, particularly the black and red varieties, is popular due to its better nutritional value. Anthocyanins and proanthocyanidins are two major flavonoid subcategories with broad physiological functions and therapeutic significance. However, pigment deposition is a complex process, and the molecular mechanism involved remains unknown. This review explores the metabolites responsible for the pigmentation in various rice tissues. Moreover, the current challenges, feasible strategies, and potential future directions in pigmented rice research are reported.

## 1. Introduction

Rice (*Oryza sativa* L.) is a major cereal crop for almost half of the world’s population [1]. Its demand continues to rise as the world’s population is projected to reach 9.7 billion by 2050 [2,3]. Rice offers diverse dietary nutrients, including carbohydrates, vitamins, and micronutrients [4,5]. Unfortunately, dehulling and milling processes remove many nutrients, particularly micronutrients, fatty acids, antioxidants, and fiber [6]. As a result, developing countries where rice is a staple food are experiencing micronutrient deficiencies, along with a significant increase in lifestyle-related diseases, such as diabetes, hypertension, and obesity [7]. Thus, there is an urgent need to produce rice with better nutritional value. To date, several bioactive compounds and micronutrient modulation processes have been employed to develop rice with superior nutritional quality for frequent consumers.

**Figure 1:**
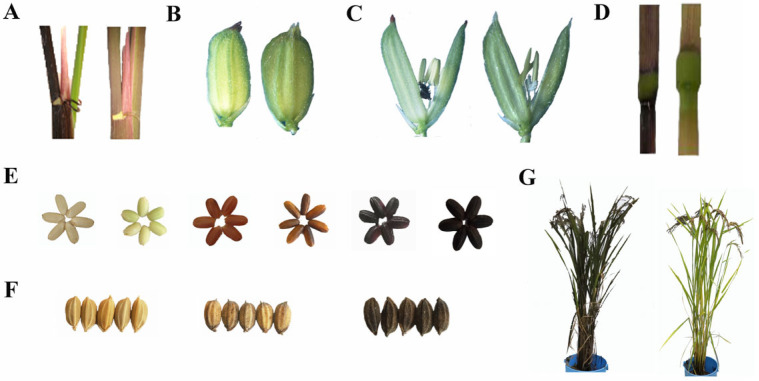
Genetic diversity involving rice pigmentation. (**A**) Leaf sheath; (**B**) apiculi; (**C**) stigma; (**D**) culm; (**E**) seed pericarp; (**F**) seed hull; (**G**) whole plant.

Pigmented rice exhibits significant genetic diversity in tissue coloration, including leaf, culm, apiculus, stigma, caryopses, and hull, with color variations ranging from dark purple to maroon to green (Figure 1) [8]. Black and red rice caryopses are rich in amino acids, functional lipids, dietary fiber, vitamins, minerals, anthocyanins, and phenolic compounds and are marketed as health-promoting foods for rice consumers [9,10]. Moreover, pigmented rice is known to offer a variety of potential health benefits, such as anti-inflammatory, antioxidant, anticancer, and hypoglycemic activities [11]. They also serve as a widely used natural food colorant [12]. Multi-omics approaches have facilitated numerous genetic and biochemical discoveries in pigmented rice cultivars [13]. Despite the widening knowledge of pigmented rice formation and function, the molecular mechanisms underlying rice pigment deposition remain to be elucidated.

In this review, we summarize the available information on the metabolites and corresponding genes responsible for grain pigment composition and highlight the challenges and strategies for future research in this field.

## 2. Identification of Metabolites in Black/Red Rice Grains

Colored grains are known for their superior nutritional value and are used as food colorants. Until recently, most studies on pigmented rice have focused on the complex relationship between bioactive compounds and antioxidants in black and red rice [14,15]. With the advancements in metabolomic technology, an increasing number of pigmented metabolites associated with pigmented rice grains have been identified [16,17]. Anthocyanins are responsible for the purple to black pigmentation in rice. Genetic diversity affects the anthocyanin content and composition in rice grains. Black rice contains approximately 18 distinct anthocyanins, with cyanidin 3-O-glucoside (C3G) and peonidin 3-O-glucoside (P3G) as the two primary anthocyanins, constituting 64–90% and 5–28% of the total anthocyanins, respectively [18,19,20,21]. Similarly, Mackon et al. (2023) employed high-performance liquid chromatography (HPLC) to examine anthocyanin content at different developmental stages of rice caryopsis [22]. They reported that anthocyanin deposition begins around 8 days post-flowering (DPF), continues from 10 to 20 DPF, and reaches a peak during the dough phase. Zhang et al. (2023) screened 12 anthocyanins, particularly cyanidin 3-O-galactoside, C3G, P3G, and cyanidin 3-O-rutinoside, all with relatively high content, using non-targeted metabolomics [23]. Furthermore, there is a complex diversity in the anthocyanin content among black rice cultivars. Proanthocyanidins, also known as condensed tannins, are responsible for red pigmentation through their oligomeric or polymerized flavan-3-ol units [24,25]. Procyanidin was identified as the major component in the bran layer of red-hulled rice [25]. Chen et al. (2016) analyzed 28 red rice varieties to systematically access the contents and proportions of proanthocyanidin oligomers and polymers in the bran layer [26,27]. The results showed significant differences in the phytochemical composition across genotypes, indicating variations in the proanthocyanidin biosynthesis among the varieties [26,27]. In addition, Sedeek et al. (2023) identified a total of 625 metabolites in 63 pigmented rice varieties, of which 375 metabolites showed significant differences in the abundance between red and black rice, indicating the genetic diversity of pigment deposition in grains [13]. Overall, there are limited reports on the anthocyanin or proanthocyanidin composition among different pigmented rice varieties, and further research is needed to elucidate these variations.

## 3. The Genetic Basis of Pigmentation

**Figure 2:**
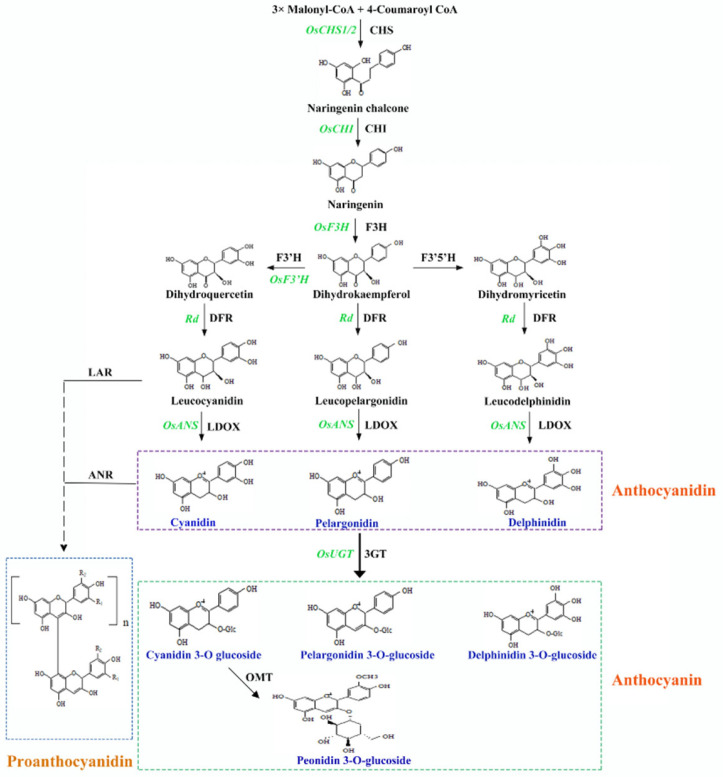
A simple illustration of the anthocyanin and proanthocyanidin biosynthetic axes. Black text indicates synthesis enzymes, and green represents corresponding enzyme-encoding functional genes. CHS, chalcone synthesis; CHI, chalcone isomerase; F3H, flavanone 3-hydroxylase; F3’H, flavonoid 3’ hydroxylase; F3’5’H, flavonoid 3’5’ hydroxylase (F3’5’H); DFR, dihydroflavonol 4-reductase; LDOX, leucoanthocyanidin oxidase; 3GT, 3-glucosyl transferase; OMT, O-methyltransferase; LAR, leucoanthocyanidin reductase; ANR, anthocyanidin reductase. The depicted biosynthetic axis supports evidence from Dixon et al. [30,34,35].

Flavonoids are common bioactive secondary metabolites found in higher plants, and they strictly regulate flowers, fruits, seeds, and other tissue pigmentation [28]. Anthocyanins and proanthocyanidins are the products of a specialized flavonoid axis (Figure 2) involving numerous structural (Table 1) and modulatory genes, whose combined action determines the pigmentation of various rice tissues (Table 2) [29]. Thus, rice pigmentation is managed by a CAP system, where “C” (chromogen) refers to structural genes, and “A” (activator) and “P” (tissue-specific modulator) refer to regulatory genes [30].

### 3.1. Anthocyanins and Proanthocyanidins Biosynthesis in Rice

Anthocyanidin biosynthesis begins with malonyl-CoA and 4-coumaroyl-CoA, in the presence of chalcone synthase (CHS) and chalcone isomerase (CHI), to produce naringenin, the precursor of many flavonoids (Figure 2). Naringenin is then converted to dihydrokaempferol by flavanone 3-hydroxylase (F3H). Dihydrokaempferol is then hydroxylated to form dihydroquercetin and dihydromyricetin through the action of flavonoid 3′ hydroxylase (F3′H) and 3′5′ hydroxylase (F3′5′H), respectively. These three dihydroflavonols undergo reduction to leucoanthocyanidins under the action of dihydroflavonol reductase (DFR). Leucoanthocyanidins are then sequentially oxidized to form anthocyanidins under the action of leucoanthocyanidin oxidase (LDOX). The resulting anthocyanidins are then glycosylated, methylated, and acylated to form anthocyanins, which display different colors [31,32]. Proanthocyanidins belong to a separate flavonoid subgroup but share several biosynthetic genes with the anthocyanin pathway [33]. To synthesize proanthocyanins, leucoanthocyanidin reductase (LAR) and anthocyanidin reductase (ANR) catalyze a reaction involving leucoanthocyanidin and cyanidin (Figure 2).

### 3.2. Identified Structural Genes in Rice

Multiple studies have identified genes that encode proteins involved in the anthocyanin and proanthocyanidin biosynthetic pathways. CHS catalyzes the conversion of 4-coumaroyl-CoA and malonyl-CoA to naringenin chalcone in anthocyanin biosynthesis. The frequency of *CHS* family genes varies significantly among the different plant species. In rice, four *CHS* gene copies have been identified (Table 1) [32,36,37,38]. CHI controls step 2 of the anthocyanin biosynthetic process, specifically the isomerization of p-coumaroyl-CoA to form naringenin. The *OsCHI* gene in rice is orthologous to *ZmCHI1*. Moreover, mutations in *OsCHI* in rice result in a golden hull and internode phenotype [39]. Lam et. al. (2022) revealed that the knockdown of *CHS*, *CHI*, and *CHIL* mutants significantly influenced the rice flavonoid pathway [37]. F3H plays a critical role in anthocyanin synthesis, and mutations in the *F3H* gene lead to variations in anthocyanin production [40]. So far, one *OsF3H* and three *F3H* homologs (*OsF3H-1*, *OsF3H-2*, and *OsF3H-3*) have been identified. *OsF3H* has been reported to have contrasting effects on rice resistance to brown planthopper and rice blast [41,42]. *OsF3H2* also encodes F3H and is responsible for wide-ranging disease resistance in rice [43]. DFR accelerates the conversion of dihydroflavonol to leucoanthocyanidin. In rice, only the *Rd* gene has been reported to modulate proanthocyanidin synthesis [44]. Furthermore, several genes encoding *F3’H*, *ANS*, and *UGT* have been annotated in rice (Table 1).

**Table 1:** A summary of structural genes that modulate anthocyanin and proanthocyanidin syntheses in rice.

Gene Name	^a^ MSU Locus	Proteins	Reference
*OsCHS1*; *OsCHS24*	LOC_Os11g32650	Chalcone synthase	[32,36,37,45]
*OsCHS2*; *OsCHS8*	LOC_Os07g11440		[37,45]
*OsCHS12*	LOC_Os07g31770		[38]
*OsCHS28*	LOC_Os11g35930		[38]
*OsCHI*	LOC_Os03g60509	Chalcone isomerase	[39]
*OsCHIL1*	LOC_Os11g02440	Chalcone isomerase-like	[37]
*OsCHIL2*	LOC_Os12g02370		
*OsF3H*	LOC_Os03g03034	Flavanone 3-hydroxylase	[42]
*OsF3H-1*	LOC_Os04g56700	Flavanone 3β-Hydroxylase	[38]
*OsF3H-2*	LOC_Os10g39140		
*OsF3H-3*	LOC_Os04g57160		
*OsF3H2*	LOC_Os04g49194	Flavanone 3-hydroxylase	[38,43]
*OsF3’H*	LOC_Os10g17260	Flavanone 3’-hydroxylase	[32]
*Rd*/*OsDFR*	LOC_Os01g44260	Dihydroflavonol reductase	[44]
*OsANS1*	LOC_Os01g27490	Anthocyanidin synthase	[32]
*OsANS2*	LOC_Os06g42130		
*OsUGT*	LOC_Os06g09240	Anthocyanidin 3-O-glucosyltransferase	[46]
*OsANR*	LOC_Os04g53850	Anthocyanin reductase	[47]

^a^ MSU: rice genome annotation project.

### 3.3. Regulatory Systems for Anthocyanins and Proanthocyanidins in Rice

#### 3.3.1. Purple-Black Pericarp

The purple-black pericarp color is highly popular among consumers, and its pigmentation is controlled by anthocyanins. Variations in pigmentation intensity among grains of this color suggest polygenic regulation. The ternary MBW complex, consisting of R2R3-MYB transcription factors (TFs), basic helix–loop–helix (bHLH) TFs, and WD-repeat (WDR) proteins, is proposed to bind promoter elements and activate the structural genes involved in the anthocyanin biosynthesis pathway [31,48,49].

*OsMYB3*, an R2R3-MYB gene, regulates anthocyanin-mediated pigmentation in black pericarp rice. The mutation of *OsMYB3* strongly reduces 19 anthocyanin metabolites and several other flavonoids in grains (Table 2) [50,51]. Several bHLH TFs have been identified in rice, particularly *Rc*, *OsB1/Ra1/Pb*, and *OsB2* (Table 2) [52,53]. Among these, the purple pericarp trait is primarily regulated by the *PURPLE PERICARPA* (*Pp*, *Prp-a*) and *Ra*/*PURPLE PERICARPB* (*Pb*, *Prp-b*) genes on chromosomes 4 and 1, respectively [53,54]. Plants that lack *Pp* but express *Pb* produce brown pericarp grains, whereas those that express *Pp* but lack *Pb* are white. On the other hand, plants that express both *Pb* and *Pp* develop a purple pericarp [2,53]. The *Pb* locus contains two genes, *Ra* and *bHLH16*. *Ra* corresponds to the *OsB1* gene, which has an *Lc* homolog in maize that regulates anthocyanin biosynthesis. The *bHLH16* gene is homologous to the *TT8* gene in *Arabidopsis thaliana*, an MYC transcription factor that regulates pericarp pigmentation [53,55,56]. Sakulsingharoj et al. (2016) reported that a 2 bp (GT) insertion in exon 7 of *OSB1* resulted in white rice [57]. Another investigation identified key activator loci for anthocyanin biosynthesis, referred to as *KALA*, which includes *Kala1*, *Kala3*, and *Kala4*, was responsible for the black pericarp phenotype [58]. Genetic and molecular analyses have shown that *Kala1* and *Kala3* correspond to *Pp* and *OsMYB3*, respectively [2]. On the other hand, *Kala4* mimics *OSB2*, which regulates multiple structural genes encoding enzymes in the anthocyanin biosynthetic pathway, including *F3H*, *DFR*, and *ANS* [59,60,61]. Moreover, two newly identified transcription factors, *OsBBX14* and *OsHY5*, reside in the nucleus, whereby they activate the transcription of genes involved in anthocyanin biosynthesis. OsBBX14 (AtBBX22) in *Arabidopsis thaliana* directly activates OsC1 or OsB2 in synergy with OsHY5 to regulate anthocyanin synthesis in black rice pericarp [62]. Despite these findings, only one gene, *OsTTG1*, encodes a WD40 protein that plays a key role in pericarp pigmentation (Table 2) [63].

**Table 2:** A summary of regulatory genes associated with anthocyanin and proanthocyanidin syntheses in rice tissues.

Locus	Allelic Locus	^a^ MSU Locus	Gene Name	^b^ CHRX	Tissues	Reference
Kala1	Pp			1	Purple pericarp	[53,54,56]
Kala3		LOC_Os03g29614	*OsMYB3*	3	Black pericarp	[50,51,58]
Kala4	Plw	LOC_Os04g47080	*OsB1*; *Ra1*; *Pb*	4	Purple leaf, sheath, internode, caryopsis	[53,54,55,56,59]
		LOC_Os04g47059	*OsB2*; *OsKala4*	4	Black pericarp; Purple leaf; sheath; apiculus; stigma	[59,60,64,65]
		LOC_Os05g11510	*OsBBX14*	5	Black pericarp	[62]
Rc	Rc-s	LOC_Os07g11020	*bHLH*	7	Light Red pericarp	[33,44,52,66]
	Rc				Red pericarp	
	rc				White pericarp	
	Rc-g				Red pericarp	[67]
	Rc^r^				Red pericarp	[68]
	Rc-gl				white pericarp	[69]
	Rc-H2				white pericarp	[70]
Chromogen		LOC_Os06g10350	*OsC1*; *OsCPL1*	6	Purple leaf sheath; apiculus; stigma; hull	[71,72,73,74]
			*OsPa*		apiculi	[75]
			*OsPs*		stigmas	[75]
		LOC_Os02g45810	*OsTTG1*	2	Stigma; leaf; pericarp; culm; panicle; root;	[63]
		LOC_Os04g52606	*SHR5-receptor-like kinase*	4	Purple leaf	[76]
		LOC_Os04g48840		4	Purple leaf	[76]
PSH1	Rb1	LOC_Os01g39430	*anthocyanin regulatory protein*	1	purple leaf sheath	[77]
PSH1	Rb2	LOC_Os01g39560	*anthocyanin regulatory Lc protein*	1	purple leaf sheath	[77]

^a^ MSU: rice genome annotation project; ^b^ CHRX: chromosome.

#### 3.3.2. Red Pericarp

The red pericarp is predominantly observed in the wild rice species (*Oryza rufipogon* L.), the ancestor of cultivated rice. Two complementary genes, namely *Rc*, which forms the basic bHLH TF, and *Rd*, which encodes the DFR protein, contribute to the red coloration in rice grains (Table 2) [7,33,44]. The *Rc*-*Rd* genotypes result in the red pericarp phenotype commonly observed in *O*. *rufipogon*. On the other hand, the *Rc*-*rd* genotypes produce brown pericarp grain [2]. *Rc* mainly determines pericarp pigmentation, with its expression masking the white pericarp phenotype. Moreover, the mutations in *Rc* alles include *Rc* (wild type), *Rc-s*, which introduces a premature stop codon leading to light red pericarp pigmentation, and *rc*, which contains a 14 bp deletion relative to the wild-type gene, resulting in a white pericarp phenotype [33,66]. Several variants have been identified that restore the wild-type red pericarp phenotype. For example, the *Rc*-*g* allele contains a 1 bp deletion located 20 bp upstream of the 14 bp *rc* deletion [67], whereas *Rc^r^* exhibits a 44 bp deletion upstream of the same region [68]. Most African domesticated rice (*Oryza glaberrima*) varieties exhibit a red pericarp, whereas white pericarp variants contain a loss-of-function *Rc* mutation. The *O. glaberrima*-specific mutation *rc-gl* introduces a premature stop codon 146 bp upstream of the *Rc-s* point mutation site [69]. Furthermore, Singh et al. (2017) identified a distinct haplotype, *Rc-H2*, which is strongly associated with the white pericarp phenotype in the Aus group of rice cultivars [70].

#### 3.3.3. Leaf

Leaf color monitoring is simple and serves as a key morphological marker in rice breeding. Aberrations in any chlorophyll biosynthetic genes, namely *OsCAO1*, *IspF*, *YGL1*, *CSP41b*, *YGL8*, *OsCOP1*, and *BC12*/*GDD1*, can result in leaves that are primarily green but readily change to yellow or pale green [78,79,80,81,82,83]. So far, only a few genes have been identified as modulators of anthocyanin accumulation. The R2R3-MYB gene *OsC1* was first identified in cultivated rice through comparative mapping between rice and maize or nucleotide sequence homology with known maize orthologs (Table 2) [71,84]. *OsC1* is a functional chromogen gene that regulates anthocyanin biosynthesis in the leaf sheath, apiculus, stigma, and hull in rice. *OsC1* null mutations result in a non-anthocyanin-pigmented phenotype [31,72]. Similarly, Qiao et al. (2021) reported that *OrC1* in wild rice enhances the expression of *OsCHI*, *OsF3H*, and *OsANS*, therefore increasing anthocyanin accumulation in leaves [73]. Overall, *OrC1* plays a significant role in anthocyanin accumulation in the purple apiculus, leaf sheath, and stigma in *indica* rice, while in *japonica* rice, it is responsible for the purple apiculus phenotype. These findings suggest that artificial selection and *C1* gene domestication are independent events in the two subspecies. Further studies have revealed that *OsC1* allelic diversity, rather than gene expression levels, regulates anthocyanin deposition [71,72,74,85]. The Pl locus consists of the *Pb* and *Pl* genes, which regulate purple pericarp and leaf pigmentation, respectively [59]. Three *Pl* alleles, *Pl^w^*, *Pl^i^*, and *Pl^j^*, have been reported to have varying degrees of control over purple leaf pigmentation [64,65]. Another study suggested *Os04g0577800* and *Os04g0616400* as candidate genes for regulating the purple leaf phenotype [76]. Lastly, two strongly associated bHLH genes, *Rb1* and *Rb2*, were identified through map-based cloning and found to play a significant role in leaf sheath pigmentation. Moreover, the overexpression of these genes considerably increased C3G and P3G accumulation in the leaf blade, leaf sheath, and pericarp [77].

#### 3.3.4. Other Tissues

Some rice varieties show pigmentation in the apiculus, stigma, hulls, and other organs, with the genes regulating these traits being expressed in a tissue-specific manner [77]. Meng et al. (2021) identified two such genes, *OsPa*, which regulates apiculi pigmentation, and *OsPs*, which controls stigma pigmentation (Table 2) [75,86]. *OsPa* and *OsPs* are strongly associated with *OsC1* and regulate the expressions and activity of *OsDFR* and other anthocyanin biosynthetic genes. Together, these genes act synergistically to produce purple pigmentation in the apiculi and stigmas, respectively. On the other hand, *IBF1* and *BBH*/*Lsi1* regulate rice hull pigmentation [87,88,89]. Sun et al. (2018) reported that *C1* (*OsC1*), *S1* (i.e., *OsB2*), and *A1* (i.e., *OsDFR*) regulate anthocyanin deposition in the rice hull [85]. Recent studies have employed map-based cloning, genome-wide association study (GWAS), and multi-omics technology to identify genomic regions and genes that regulate anthocyanin synthesis [90,91].

In summary, several studies have identified R2R3-MYB and bHLH regulators involved in tissue-specific pigmentation; however, further research is needed to fully elucidate the regulatory mechanisms controlling pigmentation in different rice tissues. A comprehensive investigation of the genetic and signaling pathways involved in pigmentation across different rice tissues can improve the management of both beneficial and undesirable traits in pigmented rice.

## 4. Concluding Remarks and Future Perspectives

### 4.1. Genetic Improvement of Pigmented Rice

**Figure 3:**
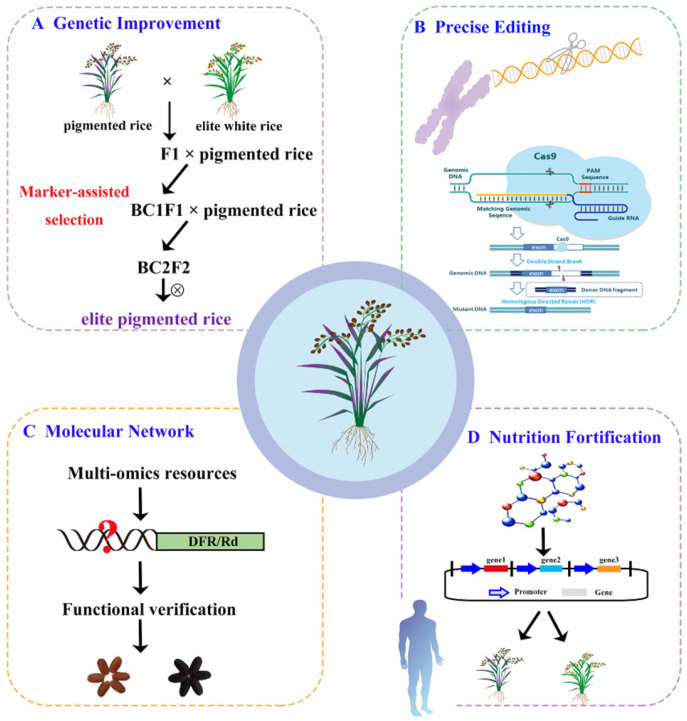
Current challenges, adaptable strategies, and potential future directions of pigmented rice research. (**A**) Improvement strategies for agronomic traits of pigmented rice. (**B**) Precise editing of linked genes through the CRISPR/Cas9 system. (**C**) Molecular mechanism analysis of pigment deposition. (**D**) Engineering of nutritional fortification for pigmented rice.

There is growing interest in the study and production of pigmented rice due to its relatively high nutritional value and associated health benefits. Most pigmented rice varieties are landraces, which often exhibit suboptimal agronomic traits and lower yields compared to white rice, as they have undergone less artificial selection [92]. Moreover, pigmented rice presents other challenges, such as longer cooking times and poor eating quality (e.g., hard texture and insufficient viscoelasticity), which, in turn, limits its production. Both agronomic traits (e.g., plant architecture, life cycle, and yield) and quality traits (e.g., cooking and eating properties) must be improved to increase the nutritional and sensory appeal of pigmented rice (Figure 3). Although it is more feasible to introduce pigmentation into modern cultivars than to improve the quality and yield of pigmented rice landraces, no studies to date have examined the effect of introducing partial pigmented rice chromosomal segments into white rice on nutrient composition. As high-coverage molecular markers have been developed for white rice, it can serve as a donor parent, and molecular marker-assisted selection can accelerate the genetic improvement in pigmented rice. Moreover, the large-scale aggregation of phenotype and genotype data, combined with genomic selection based on multiple superior alleles, can further enhance efforts to improve pigmented rice.

### 4.2. Precise Editing Through the CRISPR/Cas9 System

Genes with significant physiological roles are often pleiotropic, having multiple functions. Thus, modifying these genes may adversely affect crop production. For instance, the genes that contribute to red pericarp color are often linked with the genes that control seed shattering and dormancy [93]. Moreover, mutations in regulatory genes that increase grain yield have been shown to significantly reduce 1000-grain weight [93,94]. Genome editing technologies, particularly CRISPR/Cas9, are promising tools for improving crop traits (Figure 3). Using CRISPR/Cas9, Sedeek et al. (2023) developed a regeneration and transformation system for the efficient production of an early maturing black rice germplasm resource [13]. CRISPR/Cas9 is a powerful tool for improving agronomic traits and enhancing yields in pigmented rice cultivation.

### 4.3. Elucidating the Molecular Network Regulating Grain Pigmentation

Although various structural and regulatory genes related to anthocyanins and proanthocyanidins have been cloned, the mechanisms responsible for grain pigmentation remain unclear. Therefore, it is essential to explore the upstream and downstream regulatory elements of known genes and identify genes involved in grain pigmentation. Publicly available genomic data on pigmented rice have facilitated the screening and characterization of genes associated with grain pigmentation. Furthermore, integrated analyses involving molecular genetics, transcriptomics, proteomics, and metabolomics have revealed complex details of grain pigmentation in rice. The characterization of molecular networks is facilitated by yeast one-hybrid and two-hybrid assays, as well as chromatin immunoprecipitation (ChIP) techniques. Rice grain color intensity is affected by both environmental factors and fertilizers [8]. Further research is required to explore the relationship between grain pigmentation and environmental factors to refine the regulatory policies governing pigmented rice breeding (Figure 3).

### 4.4. Engineering of Nutritional Fortification

Rice-consuming countries are currently facing widespread micronutrient deficiencies. To address this issue, the nutritional value, quality, and yield of pigmented rice must be improved to produce a healthier variety. The genetic fortification of rice grains with functional nutrients is a primary objective of breeding programs worldwide. In recent years, the emergence of “golden rice” and “purple endosperm rice” has gained significant attention [95,96]. Tian et al. (2021) employed synthetic biology approaches to improve the riboflavin content in the rice endosperm to address riboflavin deficiency. Pigmented rice varieties are rich in flavonoids, phenols, and other bioactive compounds, making them a nutritionally superior alternative to traditional white rice [97]. Further research to improve the nutritional quality and yield of rice is important for rice-consuming populations globally, particularly in countries where rice is a staple dietary component (Figure 3).

## Data Availability

All the data used in this review paper are available online.
